# Embedded MOX-Based Volatilomic Sensing for Real-Time Classification of Plant-Based Milk Beverages

**DOI:** 10.3390/s26061976

**Published:** 2026-03-21

**Authors:** Elisabetta Poeta, Veronica Sberveglieri, Estefanía Núñez-Carmona

**Affiliations:** 1 Department of Life Sciences, University of Modena and Reggio Emilia, Via J.F. Kennedy, 17/i, 42124 Reggio Emilia, RE, Italy; elisabettapoeta@cnr.it; 2 Institute of Bioscience and Bioresources (CNR-IBBR), National Research Council URT Reggio Emilia, Via J.F. Kennedy, 17/i, 42124 Reggio Emilia, RE, Italy; estefania.nunezcarmona@cnr.it; 3 Nano Sensor System s.r.l. (NASYS), Via Alfonso Catalani, 9, 42124 Reggio Emilia, RE, Italy

**Keywords:** volatilome, smart sensing technologies, sustainability, non-destructive monitoring, food authentication

## Abstract

The increasing diffusion of plant-based milk alternatives poses new challenges at the intersection of food safety and consumer experience, particularly regarding allergen cross-contamination and beverage performance during preparation. Traditional quality control strategies are typically confined to upstream production stages and are unable to address individualized risks and sensory variability at the point of consumption. In this study, we propose an embedded volatilomic sensing approach that combines metal oxide semiconductor (MOX) sensor arrays with lightweight artificial intelligence algorithms to enable real-time, on-device decision-making. The volatilome of four commercially available plant-based milk beverages (oat, almond, soy, and coconut) was characterized using GC–MS/SPME as a reference method, while a MOX-based electronic nose provided rapid, non-destructive sensing of volatile fingerprints. Linear Discriminant Analysis demonstrated clear discrimination among beverage types based on their volatile signatures, supporting the use of MOX sensor arrays as functional descriptors of compositional identity and process-related variability. Beyond beverage classification, the proposed framework is designed to support future implementation of (i) screening for anomalous volatilomic patterns potentially compatible with accidental cow’s milk carryover in shared preparation settings and (ii) adaptive tuning of preparation parameters (e.g., foaming-related settings) in smart beverage systems. The results highlight the role of embedded volatilomic intelligence as a unifying layer between personalized risk-aware screening and sensory-oriented process control, paving the way for intelligent food-processing appliances capable of autonomous, real-time adaptation at the point of consumption.

## 1. Introduction

While food safety regulations and quality control protocols are well established at the industrial level, they are typically designed for population-scale risk mitigation and primarily operate upstream along the production chain [1]. These approaches are effective in ensuring baseline compliance and reducing systemic hazards, but they are not conceived to manage individualized exposure scenarios or the dynamic variability occurring during food preparation and consumption [2]. As a result, potential risks arising at the point of use often remain unmonitored [3]. This limitation is particularly relevant for plant-based milk beverages, whose rapid diffusion as alternatives to cow’s milk has introduced new challenges related to allergen management and product variability [4]. In this context, accidental cross-contamination with cow’s milk can pose serious risks to allergic or intolerant consumers, especially in shared preparation environments such as cafés, vending systems, and smart domestic appliances, where different beverages are processed sequentially using the same equipment [5,6].

In parallel with these safety considerations, the last decade has witnessed a growing adoption of smart food-processing appliances aimed at improving sensory performance and user experience [7]. Automated systems for beverage preparation increasingly rely on digital control of parameters such as temperature, aeration, pressure, and mixing conditions to ensure reproducibility and customization [8]. However, these systems typically operate independently of compositional safety assessment, relying on predefined recipes, barcode recognition, or user-selected settings rather than on real-time interpretation of the product being processed [9]. Consequently, safety assurance and sensory optimization remain conceptually and technologically decoupled, despite being intrinsically linked from the consumer’s perspective. To date, most smart beverage systems operate without any embedded chemical sensing layer, relying instead on predefined recipes or user-selected parameters, with no real-time verification of the product being processed [10].

Bridging food safety and consumer experience therefore requires sensing architectures capable of interpreting complex food matrices in real time and translating chemical information into actionable, on-device decisions [11]. Among the different chemical layers accessible for such real-time sensing, the volatilome represents a particularly informative interface between product composition, processing history, and functional behavior [12]. Volatile organic compounds reflect both the nature of raw materials and the technological treatments applied during production, while also influencing key physicochemical and sensory properties relevant during preparation, such as aroma release, foam formation, texture, and interaction with coffee matrices. For plant-based milk beverages, whose formulation often involves complex emulsions and stabilizing systems, volatile profiles can thus provide indirect yet meaningful information on both compositional identity and technological performance [13].

Despite its relevance, volatilomic information is still predominantly accessed through laboratory-based analytical techniques, such as gas chromatography coupled with mass spectrometry, which offer high chemical resolution but are inherently incompatible with real-time, embedded applications. Recent advances in gas sensing technologies, and in particular in metal oxide semiconductor (MOX) sensor arrays, have opened new opportunities for capturing global volatile patterns in a rapid and non-destructive manner. Rather than targeting the identification of individual compounds, MOX-based systems respond to complex mixtures of volatile species, generating characteristic signal patterns that can be interpreted as holistic fingerprints of the sampled matrix when combined with appropriate data processing strategies [14].

In recent years, several studies have investigated the use of artificial intelligence (AI) techniques combined with electronic nose (e-nose) systems for milk classification and quality assessment. Machine learning algorithms applied to gas sensor arrays have demonstrated promising performance in milk source identification, spoilage detection, and compositional grading in dairy matrices [4]. Supervised models such as support vector machines, linear discriminant analysis, and ensemble methods have been successfully employed to discriminate between different milk types and to detect freshness alterations based on volatile fingerprints. Furthermore, MOX-based sensing platforms integrated with pattern recognition approaches have been proposed for rapid monitoring of milk authenticity and quality, highlighting the potential of chemiresistive sensors for real-time dairy analysis [15,16]. However, most of these studies focus primarily on bovine milk and traditional dairy products, with limited attention to plant-based milk alternatives, whose volatilomic composition differs substantially due to botanical origin and formulation complexity [17]. In addition, many reported approaches rely on laboratory-scale instrumentation or external computational resources, rather than on embedded, lightweight intelligence suitable for integration into smart food-processing appliances [18,19].

In this context, the integration of MOX sensor arrays with lightweight artificial intelligence algorithms enables the on-device interpretation of volatilomic fingerprints, ensuring low latency, data privacy, and autonomous operation without reliance on cloud-based computation. Such embedded approaches are particularly relevant for smart food-processing environments, where rapid decision-making is required to dynamically adapt process parameters. By encoding information related to both compositional identity and functional behavior, embedded volatilomic sensing establishes the basis for simultaneously supporting real-time beverage discrimination and future point-of-use screening and process adaptation.

Recent advances in gas-sensing technologies are increasingly enabling scalable and system-integrable electronic-nose platforms, driven by progress in both device-level manufacturing and signal acquisition strategies. On the device side, wafer-level approaches for integrating high-performance nanomaterial sensing layers into MEMS architectures are emerging as key enablers for reproducible, low-power, and scalable gas-sensing chips. For example, Zhang et al. [20] proposed a wafer-level “film-first, cantilever-later” strategy enabling uniform nanomaterial incorporation (e.g., Pd/SnO_2_) into suspended MEMS structures, addressing long-standing limitations in throughput and device-to-device uniformity for micro-hotplate gas sensors. In parallel, methodological innovations in sensor operation and signal acquisition are being proposed to amplify informative transients and enhance gas identification capability in resistive sensors, moving beyond material-only optimization. In this context, Zhou et al. [21] highlighted how edge intelligence represents a key paradigm for shifting processing and inference capabilities from centralized infrastructures to the network edge, enabling real-time analysis of large volumes of data generated by distributed devices and facilitating autonomous decision-making in sensing systems.

Within this evolving technological landscape, the contribution of the present study does not lie in the development of new materials or fabrication processes, but in the definition of a system-level embedded volatilomic architecture. We demonstrate the integration of a MOX sensor array with lightweight, interpretable machine-learning models, using GC–MS–SPME as a chemical reference to validate signal interpretation. The proposed framework enables real-time discrimination of complex food headspaces under controlled conditions, translating volatile fingerprints into actionable on-device decisions. It is further conceived with scalability in mind and is compatible with future integration into smart beverage preparation appliances [22]. The aim of this work is to assess the feasibility of employing embedded MOX sensors for the real-time discrimination of plant-based milk beverages. Beyond demonstrating classification performance, the study lays the groundwork for future applications in anomaly detection, authenticity verification, and point-of-use safety screening.

## 2. Materials and Methods

### 2.1. Plant-Based Milk

**Figure 1:**
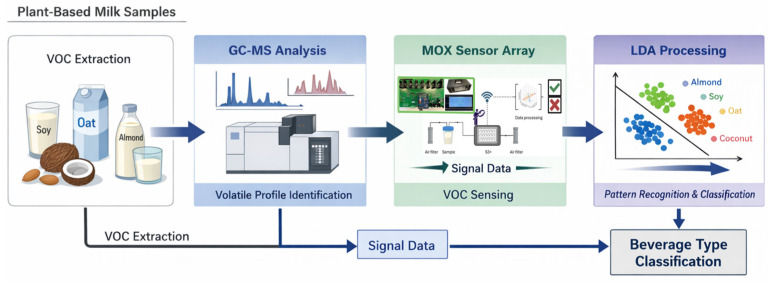
Schematic representation of the overall experimental workflow.

Four types of commercially available plant-based beverages—oat, coconut, almond, and soy—were analyzed. All samples were purchased from local supermarkets and stored under refrigeration (4 ± 1 °C) until analysis. Prior to measurement, the beverages were gently homogenized to ensure representative sampling and to minimize phase separation effects. Two parallel analytical approaches were applied for the characterization of the volatile organic compound (VOC) profiles: Gas Chromatography–Mass Spectrometry (GC–MS) and metal oxide (MOX) sensor-based analysis (Figure 1). This dual analytical setup allowed a complementary evaluation of the volatilome: the GC–MS analysis provided compound-specific identification and quantification of VOCs, whereas the MOX sensor system enabled rapid, non-destructive detection of global volatile patterns for comparative and classification purposes.

### 2.2. Gas Chromatography Mass Spectrometry Analysis

Gas Chromatography–Mass Spectrometry (GC–MS) coupled with Solid Phase Microextraction (SPME) was employed for the qualitative and semi-quantitative characterization of volatile organic compounds (VOCs) constituting the food volatilome, enabling the detection and profiling of a wide range of volatile species.

Sample preparation was performed using a DVB/CAR/PDMS 50/30 μm SPME fiber (Supelco Co., Bellefonte, PA, USA). For each beverage type (oat, coconut, almond, and soy), three replicate analyses were carried out. A 5 mL aliquot of each sample was transferred into a 20 mL glass vial sealed with a screw cap fitted with a PTFE/silicone septum. The vials were equilibrated in a thermostatic block at 40 °C for 15 min to promote headspace enrichment. Subsequently, the SPME fiber was exposed to the vial headspace for 45 min at 40 °C to allow adsorption of VOCs onto the fiber coating. The fiber was then thermally desorbed in the GC injector port at 250 °C for 6 min in direct mode to ensure complete analyte transfer. Chromatographic separation was performed using a Shimadzu GC-2020 system (Kyoto, Japan) coupled with a Shimadzu MS-QP2020 mass spectrometer. A MEGA-5MS capillary column (25 m × 0.25 mm i.d., 0.25 μm film thickness; Agilent Technologies, Santa Clara, CA, USA) was used for analyte separation. The injector temperature was set at 250 °C. The mass spectrometer operated in electron impact (EI) mode at 70 eV, with the ion source temperature maintained at 240 °C. Spectra were acquired in total ion current (TIC) mode over a mass range of 35–500 m/z, with a scan interval of 0.3 s. Hydrogen (99.99%), supplied by a GENius PF500 generator (FullTech Instruments S.r.l., Rome, Italy), was used as the carrier gas under the following conditions: pressure 35.7 kPa, column flow 2.2 mL min^−1^, linear velocity 87.4 cm s^−1^, and purge flow 4.0 mL min^−1^. The detector temperature was maintained at 240 °C, and the GC–MS interface at 200 °C. The GC oven temperature program started at 40 °C (held for 3.5 min), ramped to 90 °C at 5 °C min^−1^, increased to 220 °C at 12 °C min^−1^, and was finally held at 220 °C for 7 min.

Compound identification was performed using the instrument’s proprietary data-processing software by comparing the acquired mass spectra with the NIST11, NIST11b, and FFNSC2 reference libraries. Library matching was automatically carried out, and only identifications with a spectral similarity index ≥ 90% were accepted. When consistent matches were obtained across multiple libraries, the assignment was considered reliable [23,24]. As retention indices (RI) were not independently calculated, compound identifications should be considered tentative and based on spectral matching combined with chromatographic consistency. Retention time (RT) values obtained under the described chromatographic conditions are reported in Annex 1. Chromatographic peaks were automatically integrated using peak area as the quantification parameter, applying predefined software settings (slope 100 min^−1^, peak width 2 s, drift 0 min^−1^, doubling time (T.DBL) 1000 min), with no smoothing applied. Following automated processing, manual inspection of each chromatogram was conducted to verify peak quality and ensure correct peak assignment. Volatile compounds were quantified on a semi-quantitative basis and expressed as relative abundance (% total GC peak area), reported as mean ± standard deviation (SD). No external calibration curves or internal standards were used, as the purpose of the GC–MS analysis was to obtain comparative volatilomic profiles rather than absolute concentration values. Coupling GC–MS with SPME enabled robust qualitative profiling of complex volatile mixtures under controlled analytical conditions [25,26].

### 2.3. MOX Sensor Device and Experimental Setup

**Figure 2:**
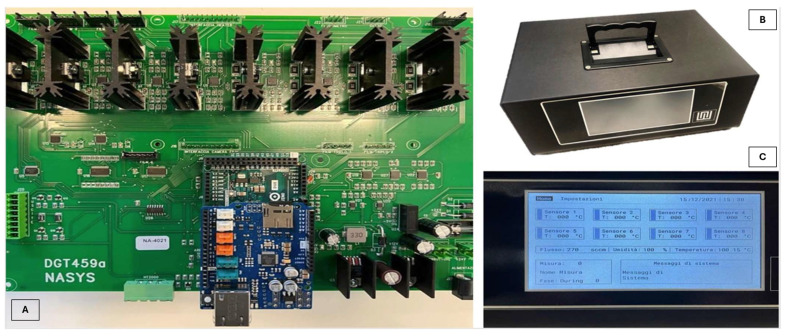
MOX-based sensing platform used in this study: (**A**) internal printed circuit board integrating the sensing elements and control electronics; (**B**) external enclosure of the S3+ device; (**C**) user interface display during operation. The system was connected to sealed glass vials for headspace sampling under controlled flow and temperature conditions.

Metal oxide semiconductor (MOX) sensors were employed for the rapid and non-destructive detection of volatile organic compounds (VOCs) emitted by the beverages. Sensor responses arise from changes in electrical conductivity upon interaction with volatile species present in the sample headspace [27,28,29,30,31]. Measurements were performed using the S3+ device, an electronic platform developed by Nano Sensor Systems (Reggio Emilia, Italy) for real-time VOC analysis (Figure 2).

The system integrates two independent MOX sensors, each comprising three sensitive elements, resulting in a total of six sensing channels (Table 1). The sensitive elements are based on tin oxide (SnO_2_), palladium-doped tin oxide (SnO_2_ + Pd), and gold-doped tin oxide (SnO_2_ + Au), a configuration that broadens the overall sensor array response toward different classes of volatile compounds. All sensors were operated at a constant temperature of 500 °C, automatically regulated by the electronic control unit to ensure measurement stability and reproducibility. Importantly, the operating temperature of 500 °C refers exclusively to the sensing layer and not to the bulk sample [31]. Each element incorporates an integrated micro-heater controlled via a closed-loop system that continuously monitors heater resistance to maintain stable thermal conditions and minimize temperature fluctuations across acquisition cycles. The beverage itself was maintained at 40 °C during preconditioning, and only gas-phase VOCs reached the heated sensing surface. Detection occurs through surface redox interactions with chemisorbed oxygen species, without implying thermal decomposition of the liquid matrix. The elevated temperature is required to activate surface oxygen species, enhance reaction kinetics, and ensure stable chemiresistive responses, in accordance with established MOX sensing principles [2].

Prior to experimental measurements, the sensors underwent a conditioning protocol consisting of thermal annealing (500–800 °C for 1–10 h) to stabilize the sensitive layers, followed by aging in clean air to optimize baseline resistance. System validation was performed through controlled exposure to calibration gas mixtures to verify response reproducibility and minimize signal drift during prolonged operation.

Measurements were conducted in 20 min acquisition cycles, including 60 s of stabilization, 120 s of sample exposure, and 60 s of recovery. Sensor signals were acquired at a sampling frequency of 1 Hz and normalized with respect to baseline resistance (R/R_0_) to ensure comparability across measurements.

The device is equipped with a measurement chamber (11 cm × 6.5 cm × 1.3 cm) designed to ensure uniform airflow and consistent sensor exposure to the sample headspace [32,33]. The fluidic system includes a membrane pump (KNF Neuberger GmbH, Freiburg, Germany; model NMP05B), an inlet solenoid valve (Camozzi Group S.p.A., Brescia, Italy; model K000-303-K11M), and an activated carbon filter for air purification, minimizing interference from ambient air [34]. Airflow was regulated up to 250 sccm, and sampling and purging phases were automatically controlled by the system [35].

For analysis, 25 mL of each beverage (oat, coconut, almond, and soy) were transferred into hermetically sealed glass containers and preconditioned at 40 °C for 10 min to promote VOC accumulation in the headspace. The containers were then directly connected to the device for sampling under controlled flow conditions. Ten measurements were performed for each beverage type, corresponding to technical replicates on the same commercial product (single brand and batch) to assess short-term repeatability under controlled laboratory conditions.

#### Machine Learning Approaches for MOX Sensor Signal Processing

The analysis of the signals generated by the metal oxide semiconductor (MOX) sensors was performed using multivariate statistical techniques and machine learning (ML) approaches, with the aim of processing and classifying the data acquired by the sensing system. Prior to data analysis, the raw sensor signals underwent a preprocessing phase to improve data quality and minimize experimental noise. Each signal was normalized with respect to its baseline resistance (R_0_), temporally aligned, and filtered using a Savitzky–Golay filter to attenuate high-frequency fluctuations while preserving the original shape of the sensor response.

Data analysis was carried out using Linear Discriminant Analysis (LDA), a supervised dimensionality reduction and classification technique. LDA was selected as a lightweight, interpretable, and computationally efficient method, consistent with the low-complexity and embedded-oriented nature of the proposed sensing framework. The method projects the original multidimensional sensor responses into an optimized discriminant space that maximizes the separation between predefined classes while minimizing within-class variability.

**Figure 3:**
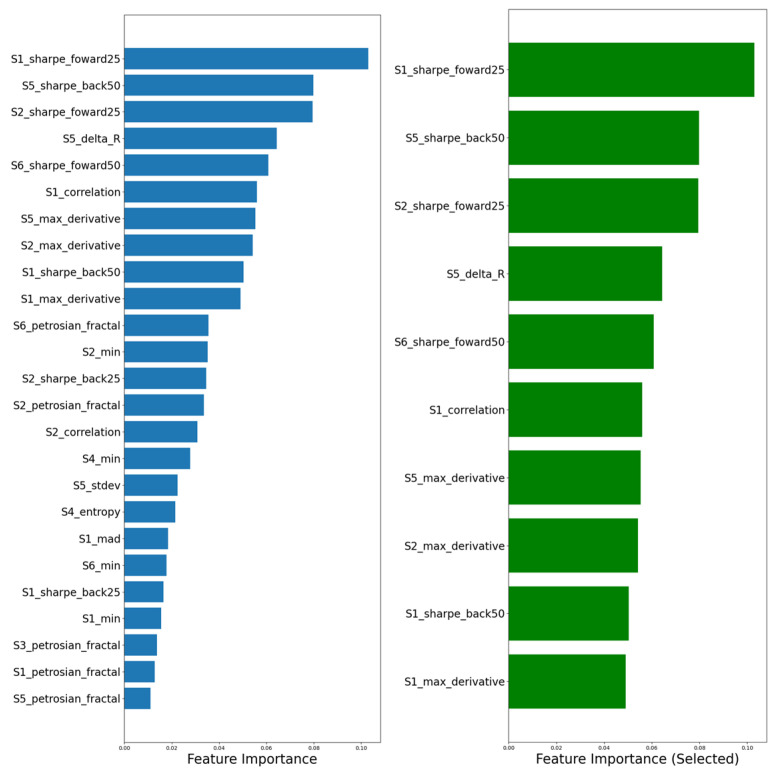
Features extracted from the recorder tracks of each sensor.

LDA was applied to the feature set extracted from the temporal responses of the six MOX sensing elements, enabling the identification of discriminative patterns associated with the different plant-based beverage types. The resulting discriminant space was used to visualize class separation and to assess the robustness and repeatability of the sensor responses across replicates (Figure 3).

All preprocessing, feature extraction, discriminant analysis, and data visualization procedures were performed using MATLAB R2019b (MathWorks, Natick, MA, USA). Cloud-based computational resources provided by the Microsoft Azure platform were used exclusively for offline data processing and visualization. However, the proposed signal processing and classification approach is inherently compatible with fully on-device implementation, as it relies on lightweight algorithms that can be deployed on embedded hardware without the need for continuous cloud connectivity.

## 3. Results and Discussion

### 3.1. Gas Chromatography Mass Spectrometry Result

**Figure 4:**
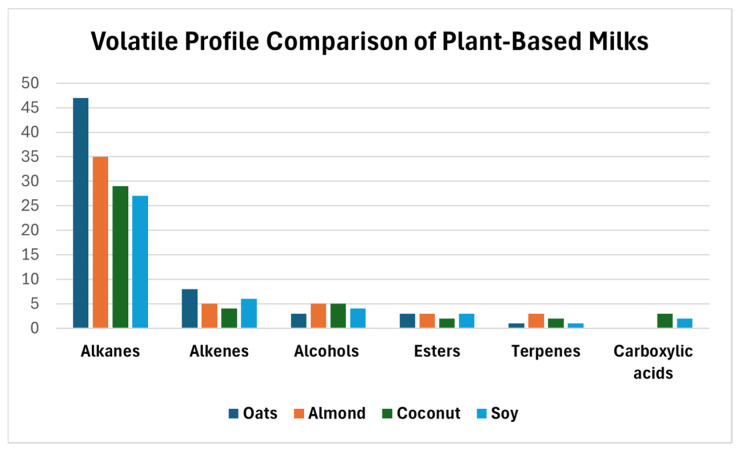
Volatile profile comparison of plant-based milks.

The GC–MS–SPME analysis of the four plant-based milk beverages led to the identification of 102 volatile organic compounds (Table 2), distributed among the main chemical classes shown in Figure 4, including alkanes, alkenes, alcohols, esters, terpenes, and carboxylic acids.

Across all samples, the volatile profiles were dominated by alkanes, which represented the most abundant chemical class in terms of relative contribution. This predominance reflects the lipid-based nature of plant-derived milk alternatives and the influence of industrial processing steps, such as thermal treatments, on the transformation of native lipid fractions.

A set of hydrocarbon compounds—including undecane 5-methyl, dodecane and its branched isomers (e.g., dodecane 4,6-dimethyl), and eicosane—was consistently detected across all beverage types. The recurrent presence of these compounds defines a common volatilomic background shared by the different matrices, indicating that despite differences in raw materials, plant-based milk beverages exhibit comparable lipid-derived volatile frameworks. This shared background provides a stable chemical baseline for comparative analysis and fingerprint-based discrimination [5].

Superimposed on this common framework, each beverage exhibited matrix-specific deviations in the relative distribution of volatile compounds. The oat-based beverage was characterized by a higher relative contribution of C10–C12 branched alkanes, with dodecane 4,6-dimethyl representing more than 22% of the total volatile fraction. This profile is consistent with the presence and processing of cereal-derived lipids. The almond-based beverage displayed a more complex volatilomic composition, enriched in C11–C14 branched hydrocarbons and characterized by the presence of octane 2,3,6,7-tetramethyl, which was scarcely detected in the other matrices. These features reflect the lipid-rich nature of almonds and contribute to a distinctive volatile fingerprint.

The coconut-based beverage showed a volatile distribution partially overlapping with that of almond in terms of alkane content, but it was clearly distinguished by the presence of oxygenated compounds, particularly 11-methyldodecanol. This compound, associated with medium-chain saturated fatty acids, emerged as a matrix-specific marker and introduced a shift toward more polar volatile species. The soy-based beverage exhibited an intermediate profile, characterized by branched alkanes similar to oat and almond, but with a relatively higher contribution of long-chain linear hydrocarbons, especially eicosane, resulting in a more uniform and less heterogeneous volatile pattern [15].

From a functional perspective, the GC–MS results indicate that the volatilome of plant-based milk beverages is largely composed of non-polar and weakly polar lipid-derived compounds, whose relative distribution varies in a matrix-dependent manner. Rather than providing isolated chemical markers, this compositional scenario generates global, reproducible volatile fingerprints that are particularly suited to pattern-recognition approaches. As such, the GC–MS analysis defines a reference volatilomic landscape against which the ability of MOX sensor arrays to capture matrix-specific volatile signatures can be evaluated.

### 3.2. MOX Sensors Result

**Figure 5:**
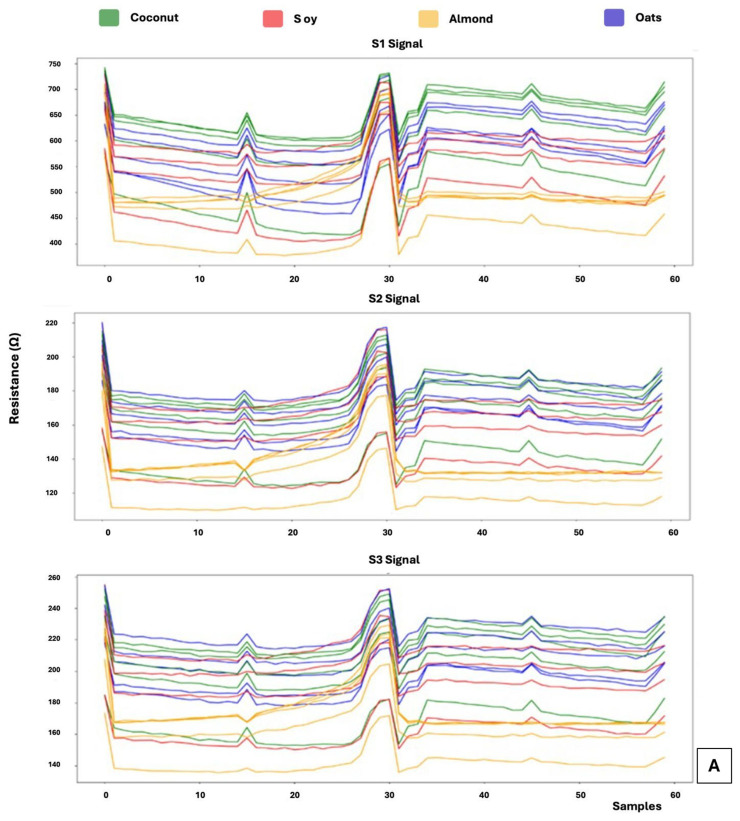

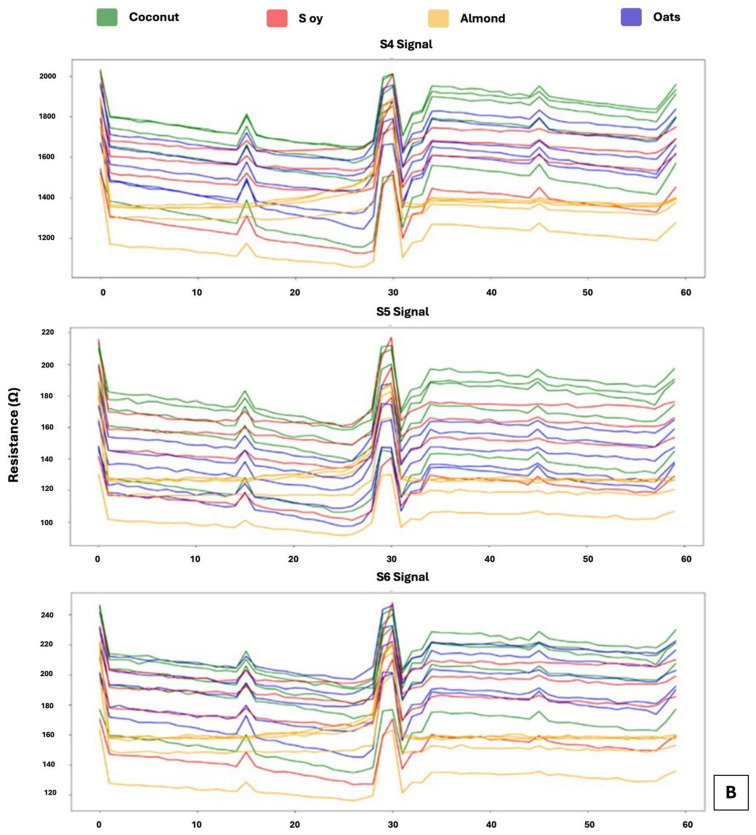
(**A**) Registered signals obtained from the analysis of the volatile fraction of plant-based milk using Sensor 1, composed of three sensitive elements (S1–S3). (**B**) Signals obtained from Sensor 2, composed of three sensitive elements (S4–S6), under the same experimental conditions.

Figure 5A,B shows the temporal responses of the six MOX sensing elements, arranged into two independent sensors with three sensitive elements each, which together constitute the sensing array of the S3+ device. During exposure to the different plant-based beverages, all sensing elements exhibited a reproducible triphasic response profile corresponding to stabilization in clean air, headspace exposure, and signal recovery after sample removal.

Consistent with the GC–MS characterization, the MOX sensor array generated distinct and reproducible response patterns for each beverage type, reflecting differences in the overall volatile composition rather than the presence of individual marker compounds. Coconut-based beverages produced broader resistance variations and slower recovery kinetics, indicating stronger and more persistent interactions between the released volatile mixture and the sensing surfaces. This behavior is consistent with the higher contribution of oxygenated compounds observed in the GC–MS analysis, which are known to exhibit stronger surface interactions with metal oxide materials.

Oat-based beverages exhibited intermediate response amplitudes combined with high signal stability across replicates, reflecting a volatilomic profile dominated by non-polar branched hydrocarbons. Soy-based beverages generated less intense responses, suggesting weaker overall interactions with the sensing materials, in agreement with the prevalence of long-chain linear hydrocarbons identified by GC–MS. Almond-based beverages displayed a distinctive and consistent response pattern, likely arising from their more heterogeneous distribution of branched hydrocarbons and matrix-specific volatile components.

The diversity of sensor responses highlights the discriminative capability of the S3+ system, which arises from the complementary sensitivity of the different sensing materials (SnO_2_, SnO_2_ + Pd, and SnO_2_ + Au). Rather than targeting individual volatile compounds, each sensing element integrates the collective interaction with the complex volatile mixture, generating a multidimensional response pattern characteristic of each beverage matrix. The high reproducibility observed across replicates confirms that these differences arise from stable volatilomic fingerprints rather than experimental variability.

**Figure 6:**
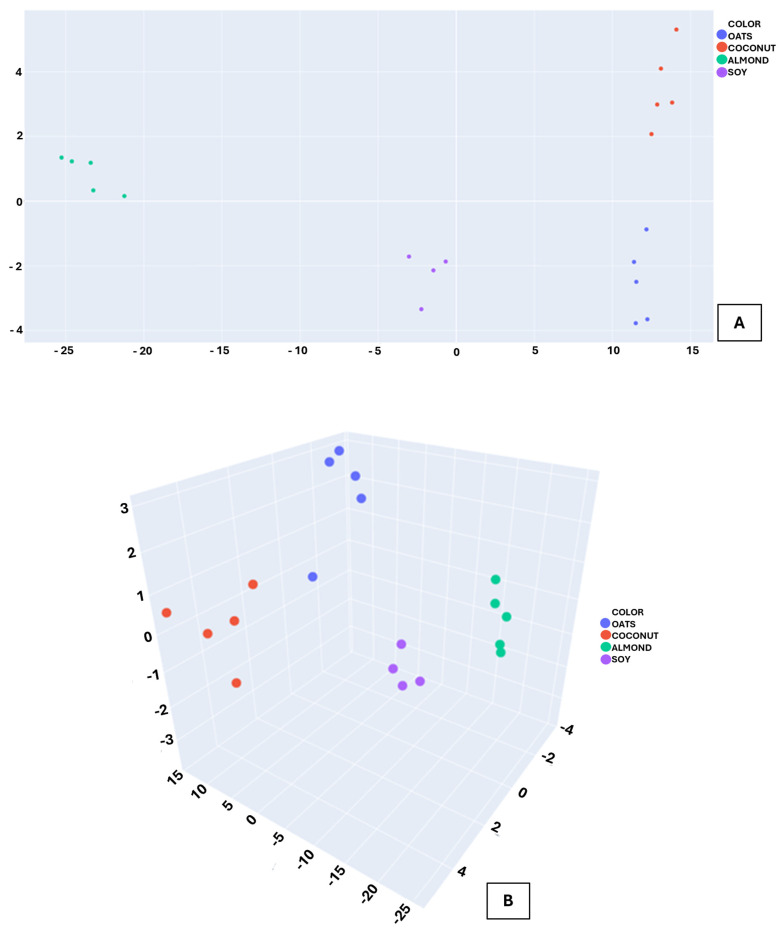
LDA score plots showing the separation of plant-based milk beverages based on MOX sensor features: (**A**) two-dimensional projection (LD1–LD2); (**B**) three-dimensional projection (LD1–LD2–LD3).

These response patterns were subsequently processed using Linear Discriminant Analysis (LDA) to project multidimensional sensor responses into a reduced discriminant space. The two-dimensional LDA projection (Figure 6A) shows well-separated clusters corresponding to the four beverage types, while the three-dimensional representation (Figure 6B) further emphasizes the spatial separation among classes and the low intra-class dispersion. Together, these results demonstrate that the discrimination capability of the system is based on robust pattern recognition within a volatilomic feature space rather than on overfitting to transient signal features.

**Figure 7:**
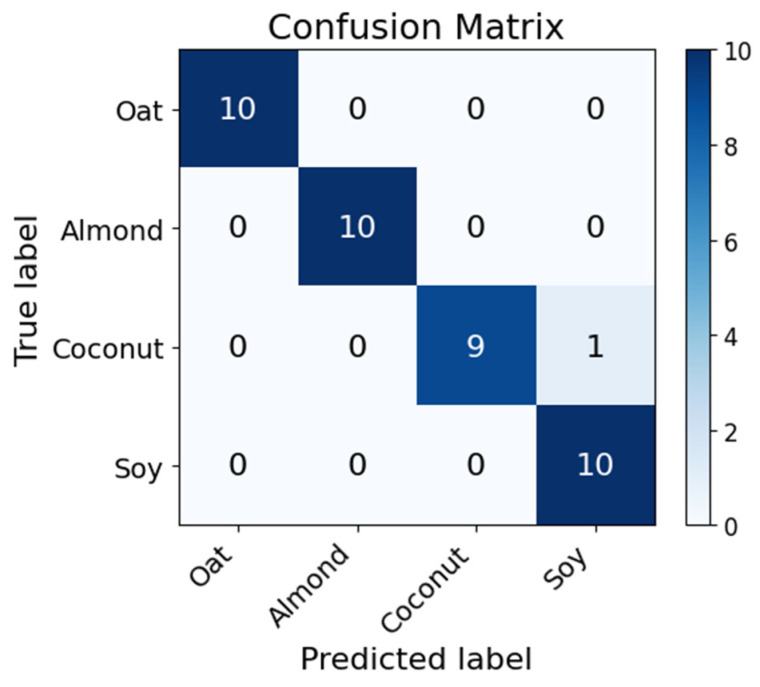
Confusion matrix obtained from the LDA classification of plant-based milk beverage samples using MOX sensor responses.

The classification performance was quantitatively evaluated using a confusion matrix and standard classification metrics. The confusion matrix obtained from the LDA model is shown in Figure 7, while the corresponding performance metrics are summarized in Table 3. The full dataset used to generate the classification results is provided in the Supplementary Materials (File S2), where the raw prediction outputs are reported.

A total of 40 samples were analyzed, corresponding to ten technical replicates for each beverage type (oat, almond, coconut, and soy). Most samples were correctly classified, with only a single misclassification observed between coconut and soy beverages. Specifically, all oat, almond, and soy samples were correctly identified (10/10), while coconut samples achieved a classification accuracy of 9 out of 10, with one sample incorrectly assigned to the soy class.

From the confusion matrix, several performance metrics were computed to quantitatively assess the robustness of the classifier. The overall classification accuracy reached 97.5%, corresponding to 39 correctly classified samples out of 40. Class-wise precision values were equal to 1.00 for oat, almond, and coconut beverages, while the soy class exhibited a slightly lower precision (0.91) due to the single misclassified coconut sample. Similarly, recall values were 1.00 for oat, almond, and soy, while coconut beverages achieved a recall of 0.90. The resulting class-wise F1-scores were 1.00 for oat and almond and 0.95 for coconut and soy, indicating balanced precision–recall performance across the dataset. When averaged across all classes, the macro-averaged metrics reached 0.98 for precision, 0.98 for recall, and 0.98 for F1-score, confirming the high reliability of the classification model.

From a system-level perspective, the proposed sensing architecture is inherently compatible with embedded and IoT-oriented implementations. The MOX sensing elements are integrated into a dedicated electronic platform (S3+) that includes the readout circuitry, micro-heater control electronics, and data acquisition modules on a compact printed circuit board. This configuration enables stable temperature regulation, real-time signal acquisition, and direct interfacing with embedded processing units. The use of lightweight machine-learning algorithms such as LDA further supports on-device implementation, allowing rapid classification without reliance on external computational infrastructure. These characteristics make the proposed platform suitable for integration into smart food-processing appliances and other edge-computing sensing systems.

Although the present study focuses on beverage-type discrimination under controlled laboratory conditions, the stability and reproducibility of the observed volatilomic fingerprints suggest that the proposed sensing architecture could be extended to additional analytical tasks, including quality monitoring and authenticity assessment. In MOX-based sensing systems, variations in volatile composition caused by oxidation processes, microbial activity, storage degradation, or matrix mixing are expected to generate measurable shifts in the multidimensional sensor response space.

In practical implementations, these applications could be addressed by training supervised models using controlled datasets, including calibrated mixture samples, dilution series, or controlled aging experiments. Such datasets would allow the system to learn characteristic deviations associated with quality degradation, freshness loss, or adulteration phenomena. Since the sensor response depends on VOC partial pressure, concentration changes may affect both signal amplitude and kinetic behavior; model robustness can therefore be improved by incorporating multi-concentration datasets and by adopting normalized or dynamic features (e.g., ΔR/R_0_, kinetic descriptors, or normalized response area). Although these scenarios were not experimentally validated in the present work, the results obtained here provide a methodological basis for future developments in volatilomic-based quality monitoring and authenticity screening.

## 4. Conclusions

This work demonstrates the feasibility of an embedded volatilomic sensing approach based on metal oxide semiconductor (MOX) sensor arrays for the real-time, non-destructive discrimination of plant-based milk alternatives. The system exploits global volatile fingerprints as functional descriptors of compositional identity and process-related variability, without aiming at compound-specific identification or allergen quantification.

The integration of MOX sensor arrays with lightweight and interpretable data processing techniques enabled robust and reproducible classification of different beverage matrices under controlled experimental conditions. Consistency with GC–MS reference results supports the use of pattern-based sensing as a suitable strategy for embedded applications, where rapid decision-making, low computational complexity, and on-device operation are required.

Beyond safety-related aspects, the proposed approach opens opportunities for adaptive control of preparation parameters in smart beverage systems, linking volatilomic fingerprints to functional properties such as foaming behavior. Overall, the results highlight the potential of embedded MOX sensor platforms as scalable and IoT-compatible solutions for the agri-food sector.

Future developments will focus on expanding the experimental framework to include (i) controlled dilution series to evaluate robustness under varying concentration levels, (ii) batch-to-batch variability assessment to strengthen model generalization, (iii) calibrated mixture experiments for the detection of cross-contamination between plant-based and dairy milk matrices, and (iv) validation under real-world operational conditions to support practical deployment in smart food-processing environments.

In addition, the integration of lightweight embedded implementation and real-time validation under operational conditions will be explored to support practical deployment in smart food-processing environments.

## Figures and Tables

**Table 1:** Configuration of the MOX sensing array integrated into the S3+ device.

Sensor Unit	Sensing Elements	Active Material	Channels
Sensor 1	Element 1	SnO_2_	1
Sensor 1	Element 2	SnO_2_ + Pd	1
Sensor 1	Element 3	SnO_2_ + Au	1
Sensor 2	Element 1	SnO_2_	1
Sensor 2	Element 2	SnO_2_ + Pd	1
Sensor 2	Element 3	SnO_2_ + Au	1

**Table 2:** Volatile compounds identified by GC–MS–SPME in the analyzed plant-based beverages. Relative abundance is expressed as the mean percentage of total GC peak area, providing an indication of the contribution of each compound to the overall volatilomic profile of the sample. Compounds not detected in a specific beverage are reported as blank cells.

RT	NOME	OATS %	ALMOND %	COCONUT %	SOY %
0.659	Acetic acid, cyano			0.08648409	0.284634079
0.829	Acetone				1.098462961
2.505	Heptane, 4-methyl	0.395646		0.81939631	
4.250	2,4-Dimethyl-1-heptene	1.83719588	1.377895	2.46802147	1.830336395
4.926	Octane, 4-methyl	0.132395985			
8.989	2,6-Dimethyl-2-trans-6-octadiene	0.277172437			
9.035	1-Butanol, 3,3-dimethyl		0.2468605		
9.176	.beta.-Myrcene			0.31466863	0.350006167
9.181	Myrcene	0.227570883	0.5145797		
9.524	Decane <n->	0.617324981	0.6029415	0.74178805	0.58478092
9.723	Heptane, 2,5,5-trimethyl	3.444765932	3.0046193	4.6540356	3.583667015
10.010	Decane, 5-methyl	0.133713819			
10.010	Octane, 3-methyl				0.276630864
10.020	Hexane, 3-ethyl-3-methyl	0.088823796		0.23368617	
10.136	1-Octene, 3,7-dimethyl	0.641714869	0.5801683	0.99001543	0.342477134
10.136	1-Undecene, 7-methyl				0.487771537
10.146	1-Pentanol, 2-ethyl	0.418730505			
10.552	1-Heptanol, 6-methyl		0.0336975		
11.040	Nonane, 5-(2-methylpropyl)	3.595290849	3.3717689	4.29773278	3.671687027
11.298	Undecane, 2-methyl			20.4841406	
11.313	Octane, 5-ethyl-2-methyl	4.648712258			4.757362442
11.493	Undecane, 5-methyl	12.04062869	24.970164		
11.493	Undecane, 6-methyl				22.0008109
12.012	Methyldodecanol				4.32827544
12.015	1-Decene, 2,4-dimethyl	3.846762039	3.5853283		2.314899678
12.139	Ethanol, 2-(dodecyloxy)			2.86052936	
12.149	1-Octanol, 2,7-dimethyl			2.30619047	1.213014992
12.311	Cymene <ortho->	0.113444279			
12.317	Decane, 5,6-dimethyl	0.193021324	0.1212932	0.3346834	0.253640443
12.323	2-Ethyl-1-butanol, pentafluoropropionate		0.0520216		
12.333	Octane, 4,5-diethyl		0.0907955		
12.809	Decane, 3,7-dimethyl	9.240751682			
13.232	Octane	4.538797144			
13.237	Octane, 2,3,6,7-tetramethyl		4.3772259	3.79412496	4.484195524
14.813	Undecane, 3-methyl			0.03721674	
15.310	Dodec-1-ene	0.098477452	0.1078216		
15.344	Pentadecane <n->	0.119265459			
15.484	Dodecane	7.368879739	2.0948844	4.30168649	
15.493	13-Dodecane		9.3695614		5.969399841
15.760	Cyclododecane		0.1464773		
15.853	Benzaldehyde, 2,4-dimethyl			0.04780862	
16.043	Dodecane, 4,6-dimethyl	22.35671089	22.822353	16.0472494	18.29821881
16.424	2-Undecene, 2,5-dimethyl	0.164546948	0.1887237		0.207870415
16.424	Heneicosane, 11-(1-ethylpropyl)				0.155868709
16.429	2-Undecene, 4,5-dimethyl-, [R*,S*-(Z)]	0.176957297			
16.515	Dodecane, 2,6,10-trimethyl	0.132918935			
16.515	Octane, 3,5-dimethyl		0.1097131	0.06204427	
16.609	Octalactone <gamma->			1.53985558	
16.618	2(3H)-Furanone, 5-butyldihydro			0.44896047	
16.675	Heptadecane	0.488938279	0.5570941	0.45901873	0.611787415
17.197	11-Methyldodecanol	2.444222233	2.9151187	4.96292775	
17.330	2-Isopropyl-5-methyl-1-heptanol	1.626872384	1.8508856	1.72577145	1.627311134
17.416	Dodecane, 1-iodo	0.904323897	0.9885177		0.749099862
17.529	Hexadecane		3.1059388		1.10993898
17.590	Tetradecane, 4-methyl			0.13741896	
17.601	2-Bromo dodecane	0.263146919	0.23674		
17.602	Hexadecane, 2,6,11,15-tetramethyl	0.16649531	0.2780139	0.23074062	0.194590086
17.776	Dodecane, 4-methyl			14.7653918	3.049507184
17.924	Dodecane, 9-methyl	1.277958187	0.5147972		
17.976	Butanoic acid, 3-oxo-, hexyl ester			0.07893163	
17.985	2,4-Dimethyldodecane	0.119677843			0.075363854
17.985	Eicosane, 2,4-dimethyl				0.067155436
17.985	Heptane, 2,4,6-trimethyl	0.152495193			
17.990	Tridecane, 5-methyl		0.1096292	0.06652137	
17.995	Undecane, 4,4-dimethyl		0.057124		
18.195	Octane, 2,6,6-trimethyl		0.1490474		
18.227	2(3H)-Furanone, dihydro-5-pentyl	0.279987402		0.83708891	0.258924939
18.229	Sulfurous acid, dodecyl hexyl ester	0.183866213			
18.232	3,5-Dimethyldodecane	0.107721713			
18.234	Nonadecane, 9-methyl		0.0544948		
18.238	Tridecane, 3-methyl		0.1029935		
18.301	Nonane, 5-butyl		0.1110585		
18.633	Tetradecane	3.444772905	3.8472263	2.17262201	3.825880896
18.973	Caryophyllene		0.0985508		
19.175	Hentriacontane <n->		0.0450301	0.09701895	
19.294	Eicosane	3.555444055	4.290375	2.61419784	5.370654214
19.395	Tricosane <n->	0.27542429	0.2906965	0.27517007	0.09535831
19.435	Humulene <alpha->	0.078523176	0.1238286		
19.503	Hexadecane, 12-methyl			0.37982424	
19.509	Methylnonadecane	2.54327426			
19.513	10-Methylnonadecane	0.177208313		0.18247149	
19.690	Nonane, 5-(1-methylpropyl)			0.88707201	
19.714	Nonadecane			0.11345004	
19.856	Heneicosane	0.199400815	0.2334827		
19.993	1-Decanol, 2-hexyl			0.13851589	
20.008	1-Dodecanol, 2-hexyl		0.2610091		
20.070	Heptadecane, 8-methyl	0.096491736		0.09087347	0.101381537
20.109	1-Hexadecanesulfonyl chloride			0.05789699	0.114731842
20.160	Decane, 1-iodo	0.209531601	0.2287515	0.94044399	0.824723837
20.375	Hexacosane <n->	0.243677742	0.121331	0.12588967	
20.375	Nonane, 5-methyl-5-propyl	0.079150716	0.0442664	0.27730044	
20.378	Tetracosane				1.066711501
20.380	5,5-Diethyltridecane		0.1926104	0.16132984	0.075870514
20.380	Heptadecane, 2-methyl		0.1036437	0.07878777	0.074955515
20.938	Benzoic acid <2-[[[4-(4-hydroxy-4-methylpentyl)-, 3-cyclohexen-1-yl]methylene]amino]-, methyl-> ester	1.280396628		1.2730052	1.340000845
20.958	Phthalic acid, bis(7-methyloctyl) ester	1.027761002	0.5417869		1.445599829
20.966	Bis(tridecyl) phthalate	0.640410482			
20.967	Bis(tridecyl)		0.71166		0.506480534
22.386	Hexacosane <n->				0.102827111
22.586	Hexadecane, 1-iodo		0.0654042		
24.476	Phthalic acid, 5-methylhex-2-yl butyl ester				0.817133333
25.269	Dodecane, 4,6-dimethyl	1.282606636			

**Table 3:** Performance metrics of the LDA classification model.

Metric	Value
Accuracy	97.5%
Precision (macro)	0.98
Recall (macro)	0.98
FI-score (macro)	0.98

## Data Availability

The original contributions presented in this study are included in the article. Further inquiries can be directed to the corresponding authors.

## Supplementary Materials

The following supporting information can be downloaded at: https://www.mdpi.com/article/10.3390/s26061976/s1.
